# Nanotechnology Addressing Cutaneous Melanoma: The Italian Landscape

**DOI:** 10.3390/pharmaceutics13101617

**Published:** 2021-10-04

**Authors:** Luigi Battaglia, Anna Scomparin, Chiara Dianzani, Paola Milla, Elisabetta Muntoni, Silvia Arpicco, Roberta Cavalli

**Affiliations:** 1. Department of Drug Science and Technology, University of Torino, 10125 Turin, Italy; luigi.battaglia@unito.it (L.B.); anna.scomparin@unito.it (A.S.); chiara.dianzani@unito.it (C.D.); paola.milla@unito.it (P.M.); elisabetta.muntoni@unito.it (E.M.); silvia.arpicco@unito.it (S.A.); 2. Department of Physiology and Pharmacology, Sackler Faculty of Medicine, Tel Aviv University, Tel Aviv 69978, Israel

**Keywords:** nanotechnology, nanoparticles, melanoma, Italy

## Abstract

Cutaneous melanoma is one of the most aggressive solid tumors, with a low survival for the metastatic stage. Currently, clinical melanoma treatments include surgery, chemotherapy, targeted therapy, immunotherapy and radiotherapy. Of note, innovative therapeutic regimens concern the administration of multitarget drugs in tandem, in order to improve therapeutic efficacy. However, also, if this drug combination is clinically relevant, the patient’s response is not yet optimal. In this scenario, nanotechnology-based delivery systems can play a crucial role in the clinical treatment of advanced melanoma. In fact, their nano-features enable targeted drug delivery at a cellular level by overcoming biological barriers. Various nanomedicines have been proposed for the treatment of cutaneous melanoma, and a relevant number of them are undergoing clinical trials. In Italy, researchers are focusing on the pharmaceutical development of nanoformulations for malignant melanoma therapy. The present review reports an overview of the main melanoma-addressed nanomedicines currently under study in Italy, alongside the state of the art of melanoma therapy. Moreover, the latest Italian advances concerning the pre-clinical evaluation of nanomedicines for melanoma are described.

## 1. Introduction

The clinical treatment of skin cancers, including cutaneous melanoma, has attracted much research interest. Of note, cutaneous melanoma is a global health issue, being one of the most aggressive cancers with a high rate of morbidity and mortality. Recent research of Bray and colleagues, considering 185 countries, reports 324,635 new cases of melanoma in 2020 [1].

Moreover, the results of epidemiological studies suggest that melanoma incidence will increase in the near future [2]. The countries with the highest risk are Australia, New Zealand, North America and Europe, together representing 85% of global incidence per year. 

It is well known that recently developed clinical practices, such as targeted therapy and immunotherapy, have been approved by the Food and Drug Administration (FDA) for the treatment of melanoma [3]. Targeted therapies exploit the use of drugs targeting specific gene alterations that are able to block melanoma oncogenesis. Currently Rapidly Accelerated Fibrosarcoma homolog B (BRAF) mutations represent the main drug target. On the other hand, immunotherapies stimulate the response of a patient’s T cells.

Despite these innovative therapeutic regimens, the clinical treatment of advanced or metastatic melanoma is still challenging. In this scenario, nanomedicine is considered a promising therapeutic strategy to improve the clinical outcomes.

This review aims at describing past and current attempts by Italian scientists to exploit their expertise in the nanotechnology field for innovative and efficient therapies addressing cutaneous melanoma. Within this concern, it should be pointed out that the originality of the approach should merge with a high translational potential, in order to be considered as a promising pharmacological therapy for clinical application. A brief overview of the available clinical treatments of cutaneous melanoma will be also described.

### 1.1. Classification, Staging, Risk Factors, Associated Deaths

Despite being the third commonest skin cancer (after basal and squamous cell carcinoma), malignant melanomas are responsible for 65% of associated deaths [4,5]. According to their body distribution, melanomas are broadly classified as cutaneous and extracutaneous, the former being the commonest type [6]. Cutaneous melanomas originate from atypical and genetically altered melanocytes [7] and according to the World Health Organization (WHO), they can be classified into four histologic subtypes [8,9].

1. Lentigo maligna melanoma (LMM) is the second commonest type [10], developing in the chronically sun-exposed skin, such as head and neck [11]. Given that LMM arises on visible areas and owing to its slowest progression rate among all histologic subtypes [12,13], it is usually diagnosed at an early stage with a good prognosis.
2. Superficial spreading melanoma (SSM) is the commonest type [14] and it usually arises on intermittently sun-exposed skin, such as on trunk and extremities [15]. Despite being endowed with a higher growth rate compared to LMM, SSM is usually associated with good prognosis [12,13].
3. Nodular melanoma (NM) consists of a smooth and uniform pigmented nodule, which predominantly spreads in the skin dermis [11]. For this reason, NM is reported to be more aggressive than the other subtypes [16].
4. Acral lentiginous melanoma (ALM) is the rarest histologic subtype [17], arising from glabrous (non-hair-bearing) skin, such as on soles and palms and nail beds [18]. ALM is associated with poor prognosis, usually because it is diagnosed at an advanced stage [19,20].

Atypical changes of a benign melanocytic lesion (nevi), which are commonly visible and well-circumscribed on healthy skin, should be carefully investigated, since they could give rise to melanoma in situ (MIS), consisting in an abnormal proliferation in the skin epidermis, without dermis invasion [21]. Nonetheless, MIS can further spread into the deeper layers of skin, acquiring metastatization potential [22,23]. Therefore, staging is the most important prognostic indicator for melanoma. Within this concern, the American Joint Committee Cancer (AJCC) Melanoma Staging System is the most accepted and periodically updated system, with the most recent edition (eighth) being released in 2018 [24]. Accordingly, as in other solid tumors, melanoma stage is established through the following criteria: tumor dimension; number of metastatic nodes; presence of distant metastases [25]. Four stages can be identified [26].

- Early stages (stages 0–II): skin localized melanoma, without spreading beyond the primary site.
- Stage III: presence of loco-regional metastasis into local lymph nodes.
- Stage IV: presence of distant metastases.

Patients diagnosed with early stage disease have a 5-year survival of 98%, whereas patients with lymph node spread have 62%, and patients at stage IV have an 18% 5-year survival. The stage IV high mortality rate is probably due to the fact that melanoma cells easily reach the bloodstream and can be transported to distal body sites [27]. As for other solid tumors, the lungs and liver are common sites of metastasis (about 10 and 10–20% of patients, respectively); however, a specific common site of metastatic spread for cutaneous melanoma is the brain (about 12–20% of patients), especially when the primary lesion is located in the head, neck or trunk. On the other side, skeletal and gastrointestinal metastases are relatively uncommon, sometimes detected in patients at late-stage disease [28].

Excessive sun exposure resulting in sunburn is an important risk factor for the development of melanoma [29,30,31], in particular in childhood and young adulthood, because of the larger number of melanocyte progenitor cells (melanoblasts) interested [32,33]. Similarly, artificial sources of UV light, such as a tanning bed, can contribute to the total UV exposure burden, with an increased incidence of melanoma diagnosed in subjects younger than 30–40 [29,34]. Epidemiological analyses have identified two main forms of cutaneous melanomas resulting from UV exposure [35,36,37].

1. Melanomas without cumulative sun-induced damage: arisen on intermittently sun-exposed skin such as trunk and proximal extremities. It is associated with early life sunburns and usually develops at a young age (<55 years) [38,39], with an intermediate UV signature mutations burden [32].
2. Melanomas with cumulative sun-induced damage: arisen on chronically sun-exposed skin such as face, ears and neck. It develops significantly later in life, typically in individuals older than 60 [40], is characterized by pre-cancerous keratinocytic lesions and solar elastosis and harbors a very high mutation burden [41].

Other risk factors include phenotype/genetic factors (UV susceptibility), frequently associated to familiar risk [42,43]; comorbidities (i.e., Xeroderma pigmentosum) [44]; organ transplantation, with medically induced immunosuppression reported to be a significant risk factor [45,46].

### 1.2. Mutational Burden

Melanoma has the highest frequency of genetic mutations among all types of cancer, mainly those that are UV light driven: it has, approximately, a median of more than 10 mutations per megabase [47,48]. According to the whole-exome sequence analysis of melanoma patients carried out by The Cancer Genome Atlas (TCGA), the following four main melanoma mutants can be identified: BRAF, Neuroblastoma Rat Sarcoma (NRAS), Neurofibromatosis type 1 (NF1) and Triple-wild-type [49]. There are many differences among the subtypes in terms of mutations burden: melanomas arisen on chronically sun-exposed skin harbor the highest numbers of mutations, especially NF1 and NRAS, and occasionally BRAF [50], while melanomas arisen on intermittently sun-exposed skin usually have an intermediate number of mutations, the commonest being BRAF V600E (50%) and NRAS (15–20%). Moreover, the mutation burden increases along with malignant evolution: invasive melanomas show Cyclin-Dependent Kinase inhibitor 2A (CDKN2A) loss, Phosphatase and Tensin Homolog (PTEN) loss, or TP53 mutations [51]. 

Identifying the genomic subtype of melanoma is an important requirement for the clinical management of melanoma patients [52]. The BRAF gene encodes for a serine/threonine kinase belonging to the Mitogen-Activated Protein Kinase (MAPK/MEK) pathway. BRAF interacts with MEK, resulting in MEK phosphorylation and subsequent Extracellular signal-Regulated Kinase (ERK) activation, which ultimately promotes cellular growth and inhibits apoptosis [53]. Mutations in BRAF result in MAPK function activation, independently from RAS upstream signaling. The BRAF gene is the commonest mutated gene in melanoma (40–50% of patients with cutaneous melanoma) [52,54,55] and 70–88% of all BRAF mutations is represented by the V600E mutation, which consists of a substitution of glutamic acid for valine in position 600 [52,54,56]. BRAF V600E mutations are essential to guide treatment decision-making: indeed, melanomas harboring BRAF V600 mutations are generally responsive to BRAF inhibitors (BRAFi) and/or MEK inhibitors (MEKi), which, therefore, are the main targeted agents for melanoma therapy [57]. 

The BRAF mutation, together with the RAS mutation, are strictly correlated with the high Extracellular Signal Related Kinases (ERK) activity in melanoma. The activation of ERK plays a crucial role in cancer development by promoting cell proliferation [53] and invasion, altering the adhesion properties of melanoma cells, also by regulating cell proliferation in the G1 phase. Furthermore, ERK activation might also be involved in chemoresistance. Taken together, these data suggest that the inhibition of ERK is a potential therapeutic approach for melanoma treatment [58,59]. Another mechanism that can be potentially targeted for therapeutic purposes is the so-called Epithelial to Mesenchymal Transition (EMT) that involves changes in marker expression, which can be correlated to melanoma’s invasiveness and progression as well as to survival outcomes. Several pathways are involved in EMT. The literature associating EMT with the metastatic process is conflicting, while consistent evidence supports a role for EMT in chemoresistance development. Indeed, EMT results in an invasive melanoma phenotype with reduced sensitivity to BRAFi. Since an EMT switch is partly regulated by the BRAF/MEK signaling pathway in normal melanocytes, BRAF/MEK inhibitors induce the opposite reprogramming in melanoma. Furthermore, EMT-driven tumor antigens modification allows immune surveillance escape, as illustrated by the role of SNAIL in the recruitment of Treg lymphocytes [60,61,62,63].

### 1.3. Clinical Management

Recent advances in the understanding of the pathophysiology and molecular pathology of melanoma have led to new effective therapies for advanced disease, as well as to programs supporting sun protection habits, greater awareness and early diagnosis, which have significantly increased patients’ overall survival [64]. Treatment options include surgery, radiation, conventional therapy (chemotherapy) and advanced therapy (targeted therapy and immunotherapy). For early stages (0–IIA), wide excision of the primary tumor is recommended. In high-risk primary cutaneous melanoma (stage IIB/C; tumor thickness >2.0 mm) and stage III, surgery is associated with adjuvant therapies [65]. Furthermore, at stage III, surgical removal of regions surrounding the metastasized lymph nodes is indicated, too. 

Before 2011, the standard-of-care treatment for IV stage melanoma was dacarbazine [66], with no improvement in survival. Temozolomide (TMZ) was employed as a second line treatment, because it can penetrate the central nervous system, allowing it to treat melanoma brain metastasis [67,68]. Recently, thanks to advances in cancer genomics [69] and immune response [70], new advanced therapeutic agents have been approved for the treatment of metastatic melanoma, especially targeted therapy and immunotherapy [71]. 

Despite the fact that melanoma is considered to be relatively radiation resistant, radiation therapy is employed to relieve symptoms in patients with brain metastases, or tumors too large for surgical intervention [65].

#### Chemotherapy, Targeted Therapy and Immunotherapy

BRAF mutated melanomas (nearly half of the total) show response to BRAF and/or MEK inhibitors, allowing the practicing of targeted therapies [57,69]. The first developed V600-mutantBRAFi was vemurafenib, 30 times more selective towards mutated BRAF compared to wild-type. Therefore, vemurafenib exhibited from partial to complete response in BRAF-mutated melanoma patients, and no response in patients with the wild-type BRAF gene [72,73]. Dabrafenib is another highly potent BRAFi, 100 times more selective for mutated BRAFV600E compared to wild type [74]. The main limitation of BRAFi is the quick development of resistance [75,76] due to MAPK pathway reactivation [77,78,79]. Therefore, in order to counteract resistance mechanisms, MEKi have been employed, including trametinib, which was approved by the FDA in 2013 for the treatment of metastatic melanoma with BRAFV600E mutations [75]. Therefore, BRAFi and MEKi combinations have become the standard-of-care treatment for unresectable or distant metastatic V600E-mutant melanoma. The three approved combination therapies are dabrafenib/trametinib, vemurafenib/cobimetinib and encorafenib/binimetinib [80,81,82,83]. 

Immunotherapy is based upon the employment of monoclonal antibodies, in order to enhance cell-mediated immunity toward cancer cell, owing to the so-called “immune-checkpoint blockade”. The first immune-checkpoint inhibitors employed were anti-Cytotoxic T-Lymphocyte-Associated-4 protein (CTLA-4) antibodies ipilimumab and tremelimumab. Afterwards, anti-Programmed Death-1 protein (PD-1) antibodies such as nivolumab and pembrolizumab underwent rapid clinical development [84]. A combination of anti-PD-1 and anti-CTLA-4 antibodies led to benefits in melanoma treatment [85]. Contrarily to targeted therapy, the mechanisms of resistance to immunotherapy are less well understood. An upregulation of Programmed Death Ligand 1 (PD-L1) expression on melanoma cells at the invasive tumor margin may be responsible for primary resistance to anti-PD-1 immunotherapy. Nevertheless, despite the great efforts spent to develop predictive biomarkers of response to immunotherapy, the ‘positivity’ for PD-L1 expression is still undefined [86]. More recently, the interplay between intratumoral genomic changes and immune response has been investigated, because some tumor-intrinsic signaling pathways were disclosed to be associated with immune exclusion. For example, PTEN loss in melanoma cells has been implicated in the exclusion of T cells from the tumor microenvironment and, therefore, a lack of response to immunotherapy [87]. Moreover, a high mutation burden has been clearly related to an improved response to immunotherapy with anti-CTLA-4 and anti PD-1 antibodies [88,89,90]. To this aim, since whole-exome sequencing cannot be practiced in clinical routine, gene panels (comprising 170–315 genes) could serve as a useful surrogate for the evaluation of the total exonic mutation burden [84].

At the moment, the choice of frontline chemotherapy is driven by patient-specific features, such as comorbidities, biochemical and clinical parameters and patient tolerance to side effects. Currently, combinations of targeted agents and immunotherapies are under investigation. Several research findings support potentially positive interactions. In particular, recent evidence supports the concept that combined BRAF-MEK inhibitors could enhance recognition of melanoma cells by the immune system, thus favoring the activity of immune-checkpoints inhibitors [91].

**Figure 1:**
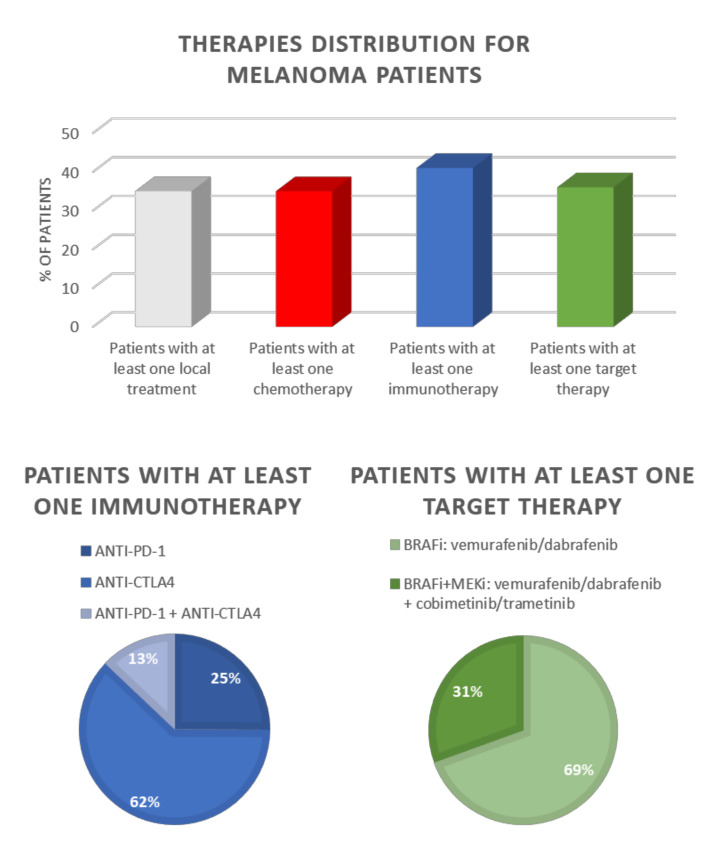
Summary of the most common therapeutic approaches in the Italian clinical landscape for the treatment of advanced metastatic melanoma. Between 30 and 40% of all patients receive at least one immunotherapy or at least one targeted therapy (upper panel). Among the patients treated with immunotherapeutic agents, Anti-CTLA4 (ipilimumab) monotherapy is the most administered (lower panel, left). BRAFi and MEKi combination is the most used targeted therapy (lower panel, right). Data elaborated from [92].

Recently, research carried out by the Italian Clinical National Melanoma Registry (CNMR) investigated the real-life clinical management data of patients with advanced cutaneous melanoma, aiming to evaluate the oncological outcome of the new therapeutic associations. The results of this research showed that immunotherapy significantly improved the patient’s survival in a real-world population. Moreover, the study pointed out that the combination of nivolumab/pembrolizumab with ipilimumab is the best therapy for the clinical management of advanced melanoma [92] (Figure 1).

Anyway, even if recent studies showed that combination therapy has a greater efficacy than monotherapy, it is associated to a higher incidence of adverse side effects.

Furthermore, while different ERK inhibitors are currently under evaluation in preclinical and clinical studies for melanoma (SCH772984, MK-8353, ulixertinib, ravoxertinib, LTT462 and LY3214996), as single agents or in combination therapy [93,94], only few innovative strategies proposed the EMT switch as a therapeutic target. Three agents (salinomycin, etoposide and abamectin) were identified through molecular screening, for targeting cells in the mesenchymal state, but to date, major evidence concerns EMT prevention and/or reversal. Although many cytotoxic agents induce EMT, inhibitors of microtubule assembly, such eribulin and the vinca alkaloids, might exert the reverse effect, as assessed in Phase III clinical trials with eribulin, used both for breast cancer and liposarcoma. Moreover, combination regimens can also be considered. Indeed, given that various Receptor Tyrosine Kinases (RTK) can mediate the EMT switch in melanoma, combinations of selected RTK and oncogenic BRAF inhibitors can be successful, such as in the case of the EMT switching inhibitor TGFßR2 with vemurafenib. Moreover, since a chronic inhibition of BRAF resulted in elevated Wnt with increased expression of the EMT inducer, WNT5A, knockdown of WNT5A was able to reverse chemoresistance caused by chronic treatment with vemurafenib [95].

Another therapeutic option for melanoma consists of topical formulations (Figure 1). Topical chemotherapy has been proposed as an adjuvant, by using imiquimod, an agent that activates Toll-Like Receptor (TLR) 7 and 8, currently employed for the clinic management of basal cell carcinoma. Activated TLR7 induces the production of different cytokines such as Interferon-α (IFN-α), Interleukin (IL)-12 and Tumor Necrosis Factor (TNF)-α, thus activating the immune system towards cancer cells [96]. Imiquimod was able to control cutaneous metastases spreading from primary melanoma [97], when used after surgical excision [98].

## 2. Rationale for Nanotechnology Approach to Malignant Melanoma

Although melanoma treatment has significantly improved in the last decade, malignant melanoma is still a major health challenge, because of its aggressive and resistant nature and its variable response to chemotherapy, which, despite being able to prolong median survival, is still to be considered as a palliative care for IV stage. Therefore, a nanotechnology approach has been proposed for the treatment and diagnosis of malignant melanoma at a preclinical or clinical level. Indeed, nanomaterials are purposed as drug delivery systems for several types of cancer, because, owing to their size and surface features, they are able to enhance targeted delivery to cancer cells, and to easily cross biological barriers. Thus, side effects in off-target tissues are reduced and efficacy is increased [99]. Additionally, nanosystems prevent the drug from chemical and/or biological degradation, and reduce drug clearance, leading to an extended half-life. Consequently, drug dosing can be reduced. 

Within this concern, a powerful nanoplatform is represented by targeted nanosystems. Among the commonest targeting moieties, antibodies allow a very selective binding to targeted cells through specific antibody-receptor interactions, while specific ligands can also be engineered for the antigens over-expressed on melanoma cells [100]. 

The most important nanosystems proposed for melanoma therapy include lipid systems (liposomes, solid lipid nanoparticles, nanoemulsions), polymeric systems (polymeric micelle and nanospheres, polymeric nanoparticles, hydrogels, dendrimers), inorganic nanoparticles (silica nanoparticles, gold nanoparticles, copper nanoparticles, nanotubes) and natural nanosystems [100]. Among the latter, exosomes (EXOs) can be included. They are cellular vesicles made of a bilayer membrane, ranging from 30 to 150 nm, carrying different types of biomolecules, including proteins, lipids and nucleic acids, with an intrinsic ability to target specific cells [101] and to overcome cell membrane and blood/brain barriers [102]. In particular, EXOs are studied as therapeutic vaccines for melanoma treatment [103,104]. Moreover, EXOs can also work as melanoma biomarkers: analyses of circulating EXOs in blood from patients could represent a promising strategy for cancer diagnosis, also in order to assess therapeutic response. Exosomal micro-ribonucleic acid (miRNA) [105], as well as several proteins [106] in circulating EXOs could be considered as possible prognostic biomarkers. Indeed, monitoring the exosomal PD-L1 level could be a relevant predictor of response to immunotherapy [107,108]. Cell-membrane-coated nanoparticles also belong to the natural category [109]. A platelet membrane coating allows melanoma cells to be targeted, whereas a red blood cells membrane coating results in macrophage clearance escape [110]. 

Finally, drug release from a nanocarrier can be controlled by internal (within the human body) or external stimuli, which are able to induce a structural modification in the nanocarrier matrix, resulting in drug release [111,112]. Internal stimuli include body temperature, pH, ionic strength and redox potential shifts. In particular, a tumor tissue shows different features compared to a healthy one: the lowest pH or the slightly higher temperature in the tumor may be exploited for a selective drug release [113]. External stimuli include light, temperature, magnetic and electrical fields and ultrasounds [114,115]. 

However, remarkable technological and toxicological drawbacks hamper the clinical translation of nanosystems to melanoma, and cancer therapy in general [116]. Indeed, the low drug payload is a relevant limitation, meaning that only potent drugs can be successfully loaded into nanocarriers, since large volumes cannot be administered to humans [117]. Moreover, stressful synthetic conditions, such as heat, extreme pH, solvents can be harmful for sensitive drugs [118], and potential contaminants, such as residual solvents, reaction byproducts and endotoxins, can lead to a considerable cellular toxicity, requiring extensive purification procedures [119]. Furthermore, rapid blood clearance, in the case of large sized nanosystems, as well as premature burst release, can reduce the tissue targeting of the loaded drugs. Within this concern, since the surface properties, along with protein corona effects, regulate the biological fate of such nanosystems, surface functionalization allows passive or active targeting to be achieved, but it is usually associated with high costs and scale-up issues [120]. Therefore, nanosystems constituted by biocompatible materials and/or physiological lipids, with a safe history of clinical use, and produced owing to feasible/easy to scale-up methods, show clear advantages for translational purposes. Nonetheless, as a matter of fact, the approval rate for novel nanomedicines is currently below 10% [121], and none of them have been specifically approved for melanoma. Indeed, it is noteworthy that, among those entering clinical trials, the major part involves the off-label use of already marketed nanotechnology products (Table 1). At present, great hopes are placed on monoclonal antibodies: after the FDA approval of ipilimumab and more recently, nivolumab, pembrolizumab and atezolizumab [3], a number of clinical trials are currently evaluating the safety and efficacy of combining two monoclonal antibodies (i.e., NCT01844505, NCT03068455, NCT03743766) [122]. Such clinical trials are not listed in Table 1, given that the table is focused on nanocarriers.

## 3. The Italian Landscape 

### 3.1. Relevant Interest of the Italian Concern

The worldwide incidence of cutaneous melanoma has increased over the last decade, up to 4–6% a year in fair-skinned populations in North America, Europe and Oceania, and recent clinical studies indicate that this increasing trend will continue in the next two decades [1,2].

**Figure 2:**
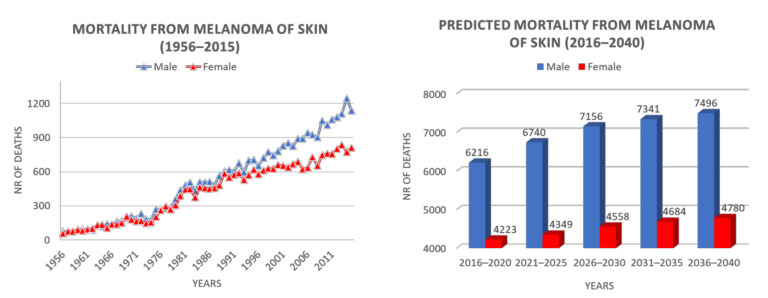
Melanoma mortality in Italy: recorded deaths in the years 1956-2015 (left panel), and prediction until 2040 (right panel). Re-elaborated from WHO IARC [128] (accessed on 15 September 21).

Incidence depends on skin pigmentation, but also varies by geographic location, among people of the same ethnicity, owing to atmospheric absorption, latitude, altitude, cloud cover and season, all parameters influencing incident UV radiation. Indeed, in 2012, the International Agency for Research on Cancer (IARC) reported an inverse latitude gradient in Europe, with a three- to six-fold higher incidence in northern than in southern countries, probably attributable to the fair skin features of northern Europe inhabitants [123]. However, among southern Europe countries, Italy showed the highest incidence (11.4 cases in every 100,000 inhabitants per year). More recent epidemiological data reported about 12,300 new cases in 2019 and 14,900 in 2020, with an increase of 2600 in 1 year [124,125]. Potential reasons for this increase could be also found in the availability of better diagnostic tools. However, in northern Italy, mortality from cutaneous melanoma is about two-fold that recorded in the southern regions, with major incidence peaks in Trieste and Genoa [126]. These data are confirmed by 2020 Guidelines of AIOM (Associazione Italiana di Medicina Oncologica) on melanoma, comprising the epidemiological data of the Italian population [127]. Cutaneous melanoma in Italy is the second cancer per incidence in the male population <50 years old. As far as concerns the female population, cutaneous melanoma is in the third position for frequency in the same age range. The melanoma incidence trend is increasing also in Italy with +4.4% per year for men and +3.1% per year for women. Epidemiological data concerning melanoma mortality in Italy are shown in Figure 2 [128]. This scenario reflects on the increasing number of Italian medical and patient associations dedicated to melanoma. While Intergruppo Melanoma Italiano is a non-profit association, constituted by over 400 medical and diagnostic specialists dealing with melanoma and non-melanoma skin cancer, including epidemiological, preventive, bio-molecular, diagnostic, therapeutic and experimental aspects [129], at least three different patients associations (Associazione Melanoma Italia Onlus, Associazione Pazienti Italia Melanoma, Associazione Italiana Malati di Melanoma) are involved in promoting assistance and social politics for melanoma patients, as well as in providing information and prevention and supporting preclinical and clinical research about melanoma [130,131,132]. 

On the other side, in Italy, the nanotechnology landscape is rapidly growing up. More than 200 structures are conducting Research and Development (R&D) in this field. Around 55% refer to public institutions and the remaining 45% to private companies. The activity is widely distributed across the national territory and usually nested around the following biggest universities and major public research organizations: Consiglio Nazionale delle Ricerche/Istituto Nazionale di Fisica e Metrologia (CNR/INFM), Consorzio Interuniversitario Nazionale per la Scienza e Tecnologia dei Materiali (INSTM), Istituto Nazionale di Fisica Nucleare (INFN), Consorzio Interuniversitario Nazionale per la Scienza e Tecnologia dei Materiali (ENEA). The major concentration is in the northern-central part of the Country, with the Lombardia region showing the highest one, with more than 20% of the structures and 30% of the employees. However, it must also be stressed that the regions with a lower number of structures do not play a secondary role, due to their high level of expertise and equipment. Moreover, during the past few years, the number of Italian private companies dealing with nanotechnology have steadily increased. Indeed, the last update of the Associazione Italiana per la Ricerca Industriale (AIRI) Nanotec IT Census 2010 identified 86 companies with activity in this field [133,134]. 

### 3.2. The Vitro/Vivo Bottleneck

**Figure 3:**
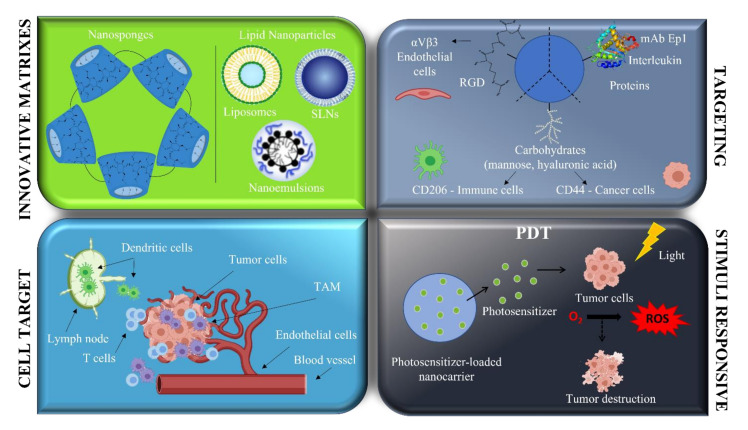
Main drug delivery strategies involving nanosystems for melanoma treatment within the Italian concern (Created with BioRender.com).

The main studies involving nanosystems aiming at melanoma treatment, developed by Italian researchers, are reported here below (Figure 3).

The major evidence concerns relevant studies on cellular models. In particular, stimuli responsive nano-platforms for potential application in PhotoDynamic Therapy (PDT) and hyperthermia to hit melanoma were developed, including photoactivable compounds, that have been conjugated either to iron oxide nanoparticles [135] or to mesoporous silica nanoparticles (MSNs) [136], or that have been encapsulated in polymeric nanoparticles [137] and amphiphilic cyclodextrin [138]. Moreover, magnetic nanoparticles mineralized with human ferritin were explored for their potential hyperthermic properties [139]. According to these studies, different melanoma cell lines (B78-H1, SK-MEL-28, B16 and A375) demonstrated a strong reduction in cell proliferation following the stimuli responsive treatment. Various nanosystems have also been proposed for adjuvant topical chemotherapy [140,141,142,143,144]. Active targeting was exploited with gold nanoparticles decorated with cyclic 4-aminoproline-RGD semipeptides, allowing internalization via receptor-mediated endocytosis, and showing the inhibition of the integrin-mediated melanoma tumor cell adhesion [145]; on the other side, lipid nanoparticles encapsulating two oncosuppressor miRNAs showed promising results on several melanoma cell lines [146].

However, the most intriguing results came from the employment of innovative materials for nanosytem formulation. Indeed, an innovative approach based on coupling nanodiamonds with a plant secondary metabolite citropten (5,7-dimethoxycoumarin) was proposed as a tool against melanoma. In vitro studies with B16-F10 cells allowed the mechanism of action of such a nanomaterial, that demonstrated higher cytotoxicity compared to the free compound, to be better understood [147]. An alternative strategy was based upon nanohydrogels for the delivery of the bovine serum amine oxidase, an enzyme that converts the polyamines overexpressed in cancer cells. In cell models, the immobilized enzyme was more active than the free one, paving the way for the use of such a nanosystem as a tool for the treatment of melanoma [148].

Nonetheless, only a few of the aforementioned nanosystems, extensively tested on in vitro cell models, shifted to the in vivo setting [149,150]. Indeed, a crucial aspect for the development of novel therapeutic approaches for melanoma therapy is the possibility to rely on animal models that recapitulate the clinical settings of the disease (Table 2). 

Murine models are often considered poorly predictable of the efficacy of the tested drugs in humans [151]. Among the syngeneic models, those based on B16 cells (in particular the B16-F10 subclone) are the most exploited to evaluate nanomedicines. Despite the skepticism arisen from the high aggressiveness of these cells once implanted in mice, as well as from the lack of genetic diversity typical of human melanoma and of BRAF mutation [230], the B16 model represents a great tool for the evaluation of traditional chemotherapies [231,232,233,234], vaccines and immunotherapies [235,236,237,238] and photodynamic/photoacoustic therapies [149,239,240] (Table 3).

Genetically engineered mouse models (GEMMs) of melanoma are a fundamental tool to evaluate the genetic components of the disease and for the development of novel targeted therapies [248], although they do not recapitulate the high mutational burden of human melanoma. Nevertheless, for instance, Rearranged during Transfection (Ret) cells inoculated in C57BL/6J mice have been used for the evaluation of dendritic cells (DC)-targeted nanovaccines [238], as the Ret model presents a high expression of regulatory T cells [249], which often limit the potency of the vaccination strategies. Furthermore, Ret melanoma cells have the capability to form spontaneous brain metastasis [250], and this represents a valuable tool to evaluate the efficacy of the proposed therapies, as in most cases of primary lesions, surgery is resolutive. Another interesting cell line is the BRAF V600E mutated D4M.3A [251], which grows in C57BL/6 mice; this model can be exploited to evaluate targeted therapies [252], as well as immunotherapeutic agents. With the establishment of immunotherapy as the gold standard for melanoma treatment, xenografts of human melanoma cell lines in immunocompromised mice became a less attractive model of the disease, although they still offer valuable insights on the evaluation of nanomedicines targeting cancer cells [241,242,243] and of stimuli sensitive-based nanomedicines [244,245]. Cells obtained by biopsies are particularly interesting, because they maintain the clinical features of the human tumor, in particular, tumor heterogeneity [248]. Once inoculated in immune-humanized mice, patient-derived tumor xenografts (PDTXs) can also be used for testing the efficacy of immunotherapeutic agents. Finally, it is worth mentioning that in the last decade, other animal models, such as zebrafish, gained great attention for the development of melanoma models. Zebrafish allow for high throughput in vivo studies [248,249,253], and furthermore, the peculiar transparency of zebrafish embryos allows for rapid visualization of fluorescently labelled melanoma cells, as well as of nanomedicines [246,247].

### 3.3. Pre-Clinical Development of Nanomedicines for Melanoma Therapy in Italy

In the last decade, in Italy, some nano-therapies for melanoma underwent an advanced pharmaceutical development, being able to reach the in vivo setting (Table 3). They were directed toward the following three main approaches: (i) nanosystems to ameliorate the pharmaceutical properties of traditional chemotherapeutic drugs, (ii) nanosystems targeting the immune system to trigger and (iii) nanosystems able to exploit PDT.

Traditional chemotherapeutics were loaded into nanosystems constituted by different matrixes. Those of natural origin are of particular interest. Among them, leukosomes are a new and very promising biomimetic nanovesicles, which combine both the physical and the biological properties of liposomes and leukocytes. Leukosomes are able to target cancer vasculature, due to the presence of the leukocyte membrane proteins responsible for cellular adhesion, such as Lymphocyte Function-associated Antigen 1 (LFA-1), Macrophage-1 antigen (Mac-1) and P-Selectin Glycoprotein Ligand-1 (PSGL-1). This formulation showed high intratumoral accumulation in mice bearing B16-F10 tumors. Furthermore, their nanosized vesicular nature allows for the encapsulation of anticancer drugs, such as doxorubicin. An in vivo tumor efficacy study in B16-F10 tumor-bearing mice showed that doxorubicin-loaded leukosomes possess a strong anti-cancer activity in terms of a reduction in tumor volume and prolonged survival [241]. Similarly inspired by nature, EXOs are nanosized extracellular vesicles that can be exploited for tumor targeting [254] and have been engineered to express TNF-related apoptosis-inducing ligand (TRAIL), a protein able to induce apoptosis in cancer cells [243]. An intratumor injection of the TRAIL-EXOs in INT12 human melanoma-bearing mice showed a strong inhibition of tumor growth, but systemic administration failed to accomplice the anticancer effect, probably due to the poor tumor homing capacity of the vesicles under study. Relevant achievements were also obtained with conventional nanosystems. The intratumor administration of cisplatin complexed with hyaluronate and loaded in fibrin gels showed promising results in mice bearing a sc human SK-MEL-28 [241]. Cisplatin encapsulated in ferritin nanoparticles, decorated with a melanoma-targeting antibody (Ep-1), also showed good in vivo anticancer activity following intravenous (iv) injection in mice bearing Colo 38 human melanoma cells tumors [242]. Other strategies, including lectin targeted dioleoylphosphatidylcholine (DOPC)/dioleoylphosphatidylethanolamine (DOPE) liposomes [255], PEGylated poly (ε-caprolactone) nanoparticles [256] and PEGylated gold nanoparticles targeted with RGD-like peptide [150], showed good ability to accumulate at the tumor site.

In order to develop immunotherapeutic agents for melanoma treatment, different types of formulations have been designed. Within this context, nanosized systems have been employed as nucleic acid vectors for specific gene delivery. Indeed, either Poly(amidoamine) (PAMAM) dendrimer decorated with the CD124/IL-4Rα peptide (targeting myeloid cells) and bearing a combination of STAT3- and C/EBPβ-specific short hairpin RNA or miRNA-142-3p [257], or cationic liposomes physically attached on the envelope of a vaccinia virus loaded with therapeutic messenger RNA (mRNA) and miRNA (viRNA) [258], showed the ability to transfect in vivo the target cells, although the anticancer efficacy still remains to be proven. Furthermore, two lipid-based formulations showed promising pre-clinicals results. A lipopolyplex vector formed by a poly-(β-amino ester) polymer (PbAE)/mRNA polyplex core, entrapped into a lipid shell composed of multivalent cationic lipid (MLV5), DOPE, distearoylphosphatidilethanolamine-polyethilenglycol (DSPE-PEG) and α -Galactosylceramide (α-GalCer) as an immunoadjuvant, has been tested as an antitumor vaccine, showing good potential to transfect DC in vivo, inducing antigen-specific CD8^+^ T cells and humoral immune responses. This activation of the immune system caused a strong anticancer effect, both inhibiting tumor growth and prolonging survival in a B16 F10 melanoma-bearing mice model [235]. On the other side, a novel clodronate-containing liposomal formulation targeting tumor-associated macrophages (TAM) was able to induce apoptosis, reduce angiogenesis and reduce the secretion of pro-inflammatory cytokines and finally reduce tumor volume in a primary melanoma model (B16-F10 inoculated s.c. in C57BL/6JOlaHsd). Moreover, the same formulation led to a significant reduction in pulmonary tumor nodules in a metastatic model of melanoma, in which lung metastases were induced by an i.v. injection of luciferase-B16-F10 melanoma cells in C57BL/6JOlaHsd mice [236].

Another interesting nano-therapeutic strategy for melanoma developed by Italian scientists is the association of photosensitizers to nanoparticles, as a tool for PDT. PDT is well established for the treatment of local tumors, since it exploits a photosensitizer as a cytotoxic agent able to destroy tumors following irradiation. Hydrophobic phthalocyanines, such as Zn(II)-phthalocyanine disulphides (C11Pc), very commonly used photosensitizers, are ideal candidates for encapsulation in nanoparticles. The use of a nanoparticulate drug delivery system allowed for an i.v. administration of the photosensitizer and promoted its accumulation into tumor tissue, thus improving its therapeutic efficacy in an amelanotic melanoma model [239]. Moreover, the conjugation of polyethylene glycol (PEG) molecules on the surface of the gold nanoparticles enhanced their water solubility and circulation time in the bloodstream, further improving the efficacy of the PDT [240]. Further interesting results have been obtained by grafting the FDA-approved photosensitizer Verteporfin to the surface of MSNs. Irradiation of B16-F10 melanoma-bearing mice following a transcutaneous administration of the verteporfin-MSN led to a cytotoxic effect and inhibited tumor neoangiogenesis [149]. PDT can also be exploited in combination with conventional chemotherapy. A Zn-based photosensitizer has been encapsulated together with docetaxel in poly(ε-caprolactone) (PCL) and poly(ethylene oxide) (PEO) “core-shell” nanoparticles. The formulation showed superior anticancer efficacy compared to controls in a model of A375 (human amelanotic melanoma cells) in nu/nu mice [244].

Lastly, some efforts were dedicated to developing diagnostic systems with potential therapeutic applications for melanoma treatment. Photo-acoustic (PA) imaging is an interesting non-invasive and highly sensitive diagnostic tool. Many of the contrast agents utilized for this technology are also cytotoxic for cancer cells. For instance, oil in water (O/W) nano-emulsions embedding hydrophobic iron-cobalt oxide cubic nanoparticles and loaded with curcumin were able to induce in vitro toxicity in melanoma cells and to accumulate in a time-dependent manner in the tumor parenchyma of melanoma-bearing mice. Although the anticancer efficacy has still to be proven, this seems a promising theranostic approach [259]. Additionally, gold nanoparticles are good candidates for PA imaging. Indeed, when loaded in Endothelial Colony Forming Cells (ECFC), gold nanoparticles are efficiently delivered to the target site (tumor) with a favorable clearance. Furthermore, such nanoparticles allowed PA signal detection and displayed anticancer efficacy following an i.v. injection in CD1 mice bearing M6 melanoma tumors [245].

### 3.4. The University of Turin Approach

It should be noticed that a relevant number of the experimental works published in this field within the Italian landscape has been carried out from researchers affiliated to the University of Turin. Indeed, since placed in Northern Italy, a region characterized by a high melanoma epidemiological incidence, as well as by a well-integrated nanotechnological research network, the University of Turin is an important incubator for the advanced pharmaceutical development of nanosystems aimed at melanoma treatment. The research developed here was focused mainly toward the following three different approaches: i) traditional chemotherapy repurposing, ii) immunotherapy and iii) a theranostic approach.

**Figure 4:**
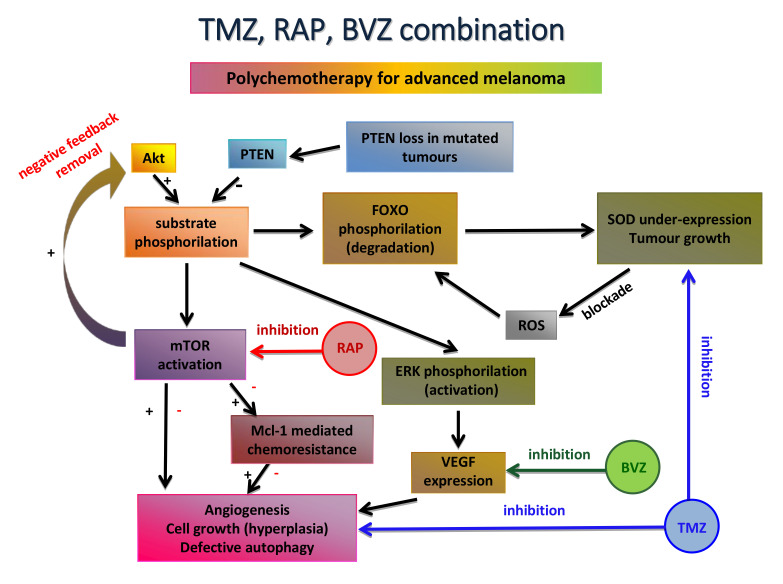
Rationale for melanoma polychemotherapy loaded in nanoemulsions. Abbreviations: Akt: protein kinase B; BVZ: bevacizumab; ERK: extracellular signal-regulated kinases; FOXO: forkhead box O; Mcl-1: Induced myeloid leukemia cell differentiation protein 1; mTOR: mammalian target of rapamycin; PTEN: phosphatase and tensin homolog; RAP: rapamycin; ROS: reactive oxygen species; SOD: superoxide dismutase; TMZ: temozolomide; VEGF: vascular endothelial growth factor.

As the first option, nanocarriers were employed in order to repurpose traditional cytotoxic drugs: improved drug delivery was achieved by increasing stability in the biological environment, by ameliorating the cross through biological barriers and by overcoming chemoresistance. Within this concern, the following three main innovative and biocompatible carrier systems, in house developed and patented, were considered: β-cyclodextrin nanosponges (CDNS), lipid nanocarriers and chitosan-shelled nanobubbles (NBs). The former one consisting of cross-linked cyclodextrins, which were used to load paclitaxel, a mitotic fuse inhibitor. The engineered formulations showed 1) an improved efficacy against several immortalized melanoma cell lines, as well as a primary culture; 2) in vitro motility and angiogenesis inhibition. In vivo experiments using B16-BL6 mouse model demonstrated that CDNS-paclitaxel reduced tumor growth and neovascularization at sub-therapeutic drug doses [232]. Among lipid nanocarriers, solid lipid nanoparticles (SLN) prepared owing to the fatty acid coacervation method were firstly explored. In particular, this solvent-free method allowed the delivery of TMZ as a dodecyl ester prodrug [233]. TMZ, a second line drug for metastatic melanoma, is administered by the oral route, and, owing to its low molecular weight, is easily absorbed by the gastrointestinal tract and able to overcome the blood–brain barrier, targeting brain metastases. However, its low stability at the physiological pH makes it necessary to use high doses to obtain therapeutic efficacy. Engineered TMZ SLN exerted promising efficacy at a sub-therapeutic dose after an i.v. administration, without exhibiting toxic effects (B16-BL6 mouse model). Interestingly, more robust results were obtained by using the same TMZ prodrug in a combination chemotherapy protocol. In this case, injectable lipid nanoemulsion for parenteral nutrition (Intralipid^®^-IL) was used as the lipid nanocarrier: mTOR inhibitor, rapamycin and the high molecular weight monoclonal antibody, bevacizumab, were used in order to reduce TMZ chemoresistance and to increase its antiangiogenic potential, respectively (Figure 4). The drug combination strongly inhibited tumor relapse, migration and angiogenesis both in vitro and in vivo, also through the activation of the immune system. No relevant side effects were noted [234]. Nonetheless, cancer chemoresistance remains a relevant issue for traditional chemotherapy. Therefore, melanoma chemoresistance can be overcome by the silencing RNA (siRNA)-mediated inhibition of the Nuclear factor E2-related factor 2 (Nrf2), which is involved in the transcription of antioxidant and cytoprotective genes. Indeed, it has been shown that upregulation of Nrf2 is able to inhibit several characteristics that confer malignant behavior to the tumor. In an interesting work, siRNA was encapsulated in aqueous droplets inside the decafluoropentane core of chitosan-shelled NBs. The siNrf2-NBs were rapidly internalized in M14 melanoma cells and induced a down-regulation of the target genes, thus sensitizing the resistant melanoma cells to cisplatin. Therefore, an siRNA-mediated Nrf2 inhibition could be a useful approach to overcome drug resistance [260].

However, the most intriguing results came from the immunotherapeutic approach. Indeed, nanosystems can be exploited for the delivery into nanocarriers of costimulatory molecules related to immune checkpoints, which mediate signals associated with the anti-tumor response. Inducible Co-Stimulator (ICOS) is mainly expressed by activated T cells and binds ICOS-ligand (ICOS-L or B7H) expressed by several immune cell types, as well as by fibroblasts. By using a soluble form of ICOS, called ICOS-Fc, developed and patented in house [261], cell adhesion and migration inhibition was demonstrated in several immortalized melanoma cell lines, as well as in a primary culture expressing ICOSL. Furthermore, ICOS/ICOSL interaction inhibited lung metastasis in a B16-F10 mouse model. The anti-tumor effect was probably exerted by acting on both cancer cells and the tumor microenvironment, including DCs, endothelial cells (ECs) and TAM. However, no effect on primary tumor growth was demonstrated, regardless of the dose, administration route and animal experimental model. An inefficient in vivo biodistribution of ICOS-Fc could explain these unsatisfactory results [262]. Therefore, the above mentioned CDNS and poly (lactic-co-glycolic acid) (PLGA) nanoparticles were used as ICOS-Fc delivery systems. Interestingly, these formulations inhibited tumor growth acting on different mechanisms. Indeed, CDNS/ICOS-Fc inhibited T regulatory lymphocytes (T_reg_), reducing IL-10 and Forkhead box P3 (FoxP3) expression [263], as immune checkpoint inhibitors do, while PLGA/ICOS-Fc nanoparticles worked efficiently despite the absence of an immune modulating effect. Furthermore, both the formulations inhibited tumor vascularization, as well as the adhesion and migration of the tumor cells expressing ICOSL [237]. Further efforts are currently underway in order to determine the molecular basis underlying the different mechanism through which these different nanocarriers exert their therapeutic effect. A different approach to exploit the immune system to fight melanoma involved the development of vaccines based on polylactic acid (PLA)/PLGA nanoparticles decorated with mannose residues, for DCs targeting. Such nanoparticles were able to encapsulate two different melanoma antigens (MART-1 MHCI and MHCII restricted peptides), together with adjuvants and immunopotentiators (mainly TLR agonists). The immunization of tumor-bearing mice with the nanosized vaccine showed superior ability to elicit a specific anticancer immune response compared to the co-administration of all the vaccine components (antigens, adjuvants, etc.) in solution. Furthermore, the treatment of C57BL/6J mice bearing either RET or B16-F10 s.c. murine melanoma with the nanovaccine in combination with immunocheckpoint modulators (PD-1 and OX40) and with ibrutininb, an inhibitor of myeloid-derived suppressor cells (MDSC), led to a strong therapeutic effect [238].

Finally, nanosized delivery systems have been exploited for diagnostic purposes. A new amphiphilic chelate of Gadolinium, GdDOTAGA(C_18_)_2,_ was synthesized and then embedded in either liposomes or dendrimersomes [264]. The latter formulation has shown optimal features to enhance the imaging capacities of the Gadolinium complex. Following the i.v. injection of the dendrimersomes in the tail vein of C57BL/6, no cytotoxic effects were reported. Furthermore, the formulation shows thermodynamic stability, water exchange rate and high relaxivity, which are fundamental for in vivo MRI imaging and for potential theranostic applications.

## 4. Current Challenges and Future Directions

Despite the introduction in clinical practice of the BRAFi/MEKi and of the immune checkpoint modulators for the treatment of metastatic melanoma, the 5-year overall survival remains poor [27]. In fact, BRAFi are effective in up to 50% of melanoma patients, but typically after 6–8 months of treatment, drug resistance phenomena lead to a relapse of the disease [265]. Immune checkpoint inhibitors are indeed more effective in achieving long-term remission but are still limited to 30% of melanoma patients as single therapy. Nevertheless, severe side effects often lead to treatment discontinuation [266].

Therefore, the main open challenges in melanoma treatment are (1) to improve the selectivity of anticancer drugs for tumor cells and the microenvironment, while sparing healthy tissues; (2) to overcome the chemo-resistance, since malignant melanoma is notoriously resistant to radiotherapy and chemotherapy, this fact being relevant to its clinical outcome. 

In this context, the targeting of tumor tissue by nanomedicines is a promising approach. Indeed, nanocarriers’ unique features (such as reduced size, variable shape, high surface area-to-volume ratio, favorable drug release profile and targeting features) promote their preferential accumulation in tumor tissues, where they can deliver multiple therapeutic agents, not only to enhance their therapeutic effect on a synergistic or additive basis, but also to overcome the acquired resistance to single chemotherapeutic drugs. Indeed, among the large number of nanomedicines in the clinical stage of development, very few are intended for melanoma therapy. Nevertheless, at the preclinical level, many nano-sized formulations showed efficacy in animal models [267].

In particular, researchers in Italy employed nanocarriers to deliver several types of therapeutic molecules, both conventional, targeted and/or immunotherapies. Despite the fact that most of these drugs are not currently used in the clinical routine for melanoma, the improved efficacy of these nanomedicines was documented in cell and animal models, in terms of therapeutic dose reduction and selective tumor accumulation, as compared to free drugs. This opens future perspectives for the repurposing of such compounds also for melanoma therapy. In fact, the future of nanomedicine will improve the efficacy of conventional therapies by exploiting the concept of personalized therapy as a consequence of the opportunity of modulating the various parameters of nanosystems. For instance, their application for the combined therapy of tumors (simultaneous delivery of multiple anticancer drugs/combination of conventional chemotherapeutics with other treatment modalities), as well as the delivery of anticancer drugs in association with photosensitizing agents, nucleic acids, antiangiogenic compounds may all better exploit the versatility of the proposed systems and their ability to overcome chemoresistance mechanisms, thus increasing the final anticancer effect [268]. Finally, bio-nanotechnology has added a new dimension to the development of nanomedicines. If nanocarriers based on supramolecular assemblies can be intelligently designed to exploit physiological or biochemical features of infectious or malignant diseases, it should be possible to carry large payloads of the respective drug to the pathogenic site. It is noteworthy that, since the vitro/vivo bottleneck is the main obstacle to pharmaceutical development, toxicity and pharmacokinetic issues should be addressed at an early stage, when selecting promising new nanomedicines, since in vivo studies will primarily decide their fate [269].

Nevertheless, although selective targeting of nanomedicines represents a great improvement in comparison to free drugs, it is a very complex mechanism and represents a challenge itself. Indeed, for instance, overexpression of a specific surface protein is not enough to assure selective targeting, as they are also normally expressed in normal cells. Therefore, in the case of unexpected and unwanted off-target distribution, the high toxicity of most of the administered drugs for melanoma therapy is still a critical point. Some studies developed in small animal models showed promising results, but currently, the translation from animal results into clinical success has been limited, since more clinical data are needed to fully comprehend the mechanism and toxicity of such nanomedicines [270]. Within this concern, pharmaceutical companies should face high expenses for manufacturing processes and clinical trials with uncertain perspectives, due to the low success rates of novel nanomedicines. Perhaps, focusing on more specific indications for novel nanomedicines, suitable for particular categories of patients affected by melanoma, as well as extensions to other types of cancer potentially sensitive to such therapeutic approaches, could be a good recommendation to maintain a profitable economic growth rate [271]. 

## 5. Conclusions

This review pointed out that nanomedicine can be a significant tool for improving melanoma treatment. Indeed, employing a nanoplatform would allow some limitations of current melanoma therapy, such as cancer resistance and lack of specificity to tumor cells, to be overcome. Moreover, nanosystems can be designed to modify the drug biodistribution, to deliver chemotherapeutics into the tumor microenvironment and to increase drug retention at the target tumor site. These benefits might allow the administration of decreased doses of chemotherapeutic compounds, thus reducing the occurrence of adverse side effects, and resulting in an improved quality of life for patients. However, due to the inherent limitations of nanotechnology, the approval rate for novel nanomedicines is below 10% and the most valuable nanotechnology approaches currently under evaluation in clinical trials for advanced melanoma involve the off-label use of already marketed nanotechnology products.

The interest of the Italian research groups for the treatment of malignant melanoma is due both to epidemiological and industrial reasons, and it led to the development of innovative nanomedicines that are able to store and release small molecules as well as biomacromolecules. Such nanodelivery systems might be employed to overcome the critical points of either traditional monotherapy or combination therapy, including the immunotherapy approach. A relevant number of them were able to overcome the vitro/vivo bottleneck, since their melanoma-fighting potential was shown in animal models, and the encouraging results obtained could pave the way for future clinical translation. Indeed, presently, all the developed nanoformulations can represent an interesting and challenging library for clinical researchers, addressing the main therapeutic issues of local advanced stage and metastatic stage melanoma.

## Figures and Tables

**Table 1:** Nanotechnology products under clinical evaluation for melanoma [122].

Product	Description	Phase	Recruitment	Trial Number
TREATMENT				
Abraxane (Celgene)	Albumin-bound paclitaxel	Phase III, monotherapy	Completed	NCT00864253 (Metastatic Melanoma)
		Phase II, monotherapy	Completed	NCT00093119 (Metastatic Melanoma)
		Phase II, monotherapy	Completed	NCT00081042 (Metastatic Melanoma)
		Phase II, monotherapy	Completed	NCT00738361 (Intraocular Melanoma)
		Phase I, Hepatic Arterial Infusion	Completed	NCT00833807 (Liver Metastasis)
		Phase II, in combination with ipilimumab	Completed	NCT01827111 (Metastatic Melanoma)
		Phase II, in combination with Bevacizumab	Completed	NCT00462423 (Metastatic Melanoma)
		Phase II, in combination with Bevacizumab o ipilimumab	Completed	NCT02158520 (Metastatic Melanoma)
		Phase II, in combination with rituximab	Recruiting	NCT02142335 (Metastatic Melanoma)
		Phase I, in combination with cisplatin, Temodar (temozolomide), interferon alfa-2b and interleukin-2 (IL-2)	Completed	NCT00970996 (Metastatic Melanoma)
		Phase II, in combination with sorafenib	Completed	NCT00483301 (Metastatic Melanoma)
		Phase II, in combination with carboplatin	Completed	NCT00404235 (Metastatic Melanoma)
Marqibo (Spectrum)	Liposomal vincristine (non-PEGylated)	Phase I/II, monotherapy	Completed	NCT00145041 (Metastatic Melanoma)
		Phase II, monotherapy	Completed	NCT00506142 (Metastatic Melanoma)
2B3-101	Doxorubicin glutathione-pegylated liposomes	Phase I/II, monotherapy or in combination with trastuzumab	Completed	NCT01386580 (Metastatic Melanoma)
IMAGING				
124I-cRGDY-PEG-dots	Silica nanoparticles with an NIR fluorophore, PEG coating and a ^124^I radiolabeled cRGDY targeting peptide	Phase I/II	Recruiting	NCT02106598 (Head/Neck Melanoma)
CANCER VACCINE				
Lipo-MERIT (Biontech RNA Pharmaceuticals)	Four naked RNA-drug products formulated with liposomes	Phase I	Active, not recruiting	NCT02410733 (Malignant Melanoma)

Abbreviations: NIR: Near Infra-Red; PEG: polyethylene glycol; RNA: ribonucleic acid.

**Table 2:** Animal models in melanoma research.

Models			Advantages/Drawbacks	References
Xenografts	Human cell lines	Nude mice	Easily available and propagated after subcutaneous transplantation. Melanoma cell lines established under non-physiological conditions for several years may result in selection of clones that differ significantly from the originating cells and are no longer representative of the original tumor. Poorly predictive of clinical outcome: drugs showing efficacy in this model often fail in clinical trials. Growth in inadequate tumor microenvironment, including the lack of an immune system.	[151,152,153,154,155,156,157,158,159]
	PDTXs	Immunosuppressed mice Nude athymic (nu/nu) mice SCID mice NSG mice	Good availability and affordability Good representation of a comprehensive patient population with different mutation burden. Helpful in guiding clinical management of the patient’s tumor: the process from target identification to validation and then to efficacy screening can be rationalized around the same model, from the patient to the mouse and then back to the patient. Useful to determine mutations required for melanocyte transformation and melanoma cell invasion. Technically challenging and time-consuming process: time for palpable tumor to develop typically ranges from three to nine months, and in many cases, tumors fail to develop. Failure in modelling immune responses: tumors do not grow in the context of an intact immune system.	[151,160,161,162,163,164]
UVR induced		HGF⁄SF transgenic mice	Useful for simulating the natural progression of melanoma development as it occurs in human beings. Strong epidermal component, or junctional activity, with a variety of histopathologies .Neonatal UVR irradiation sufficient for induction of junctional melanoma. Exposure of neonatal animals to UVR resulting in the development of lesions resembling RGP/ VGP melanoma and invasive melanoma with junctional and dermal components. Adult UVR irradiation unable to initiate melanoma but able to increase the multiplicity of melanocytic lesions in neonatally irradiated animals. Progression from early proliferative lesion to metastasis, such as that observed for k4a deletions in humans. Useful to determine the most implicated UVR band in melanomagenesis. Difference between localization of melanocytes within the mice and human skin. Spontaneous melanoma in nearly 22% of HGF/SF transgenic mice with a mean onset of 15,6 months	[165,166,167,168,169,170,171]
Chemically induced		Mice DMBA induced TPA induced	Fully functional immune system (useful for immunotherapeutic strategies). Used in combination with other models to decrease the latency of developing melanoma. DMBA alone can induce nevi in pigmented mice, useful to study mechanism(s) of malignant transformation Lack of clinical relevance to the human disease	[172,173,174,175,176,177,178]
Syngeneic		Harding-Passey cells in BALB/c × DBA/2F1 mice S91 cells in DBA/2 mice B16 cells in C57BL/6 mice	Due to melanin production, useful to study the effects of melanin content on the metabolic function of melanoma. Intact immune system. No spontaneous metastases.	[171,172,179,180]
GEM	CDKN2A loss	Mice	Loss of CDKN2A locus located at 9p21 encoding two well-identified tumor suppressor proteins, p16INK4A and p14ARF (p19ARF in mouse) Melanomas predominantly originating in the eyes, skin melanomas infrequent and mostly benign. Ink4a or ARF loss not enough to trigger melanoma development but makes animals susceptible to UVR or carcinogen-induced melanomagenesis.	[181,182,183,184,185,186,187,188,189,190,191]
	RAS mutated	HRASV12G mice UVR/DBA induced HRASV12G mice/p16INK4a/p19ARF knockout mice (Cross breeding)	Tyrosinase-driven expression of activated HRASV12G not able to trigger spontaneous melanoma development. Induction of melanoma in a relatively short latency with concurrent UVR or DMBA treatment. A new model with the capability of developing a large number of spontaneous cutaneous melanomas with a shorter latency obtained by crossbreeding between HRASV12G and p16INK4a/p19ARF knockout mice	[192,193,194,195,196]
	PTEN loss/BRAF mutated	Mice	Tyr:Cre-ERT2 transgenic mice useful to investigate BRAF V600E mutated and PTEN deleted melanomas.	[197,198]
	RCAS/TVA system	Mice	Multiple genetic alterations introduced, through retroviral-vector delivery systems, rapidly and in a sequential manner, without the requirement of crossing multiple mice strains Rapid assessment of newly identified genes on disease progression and maintenance. Well mimicked tumor microenvironment, as cancer develops from few modified surrounded by normal cells.	[151,172,199,200]
	RET controlled	Mice	Stepwise melanoma development, RET expression driven, under the control of metallothionein-1 promoter. No tumors for several months after birth, followed by growth of multiple benign melanocytic tumors that eventually become malignant and metastasize to distant organs.	[151,199,200,201]
	GRM1	Mice	Melanocyte-specific expression of GRM1 via Dct promoter able to trigger development of spontaneous, highly pigmented melanomas in skin, eyes and ear of the animals with 100% penetrance. Useful as a spontaneous uveal melanoma model.	[202,203,204]
	GNAQ mutated	Mice	When crossed with mice defective in p16INK4A and p19ARF genes, 50% of the mice developed cutaneous melanoma with a latency of ~35 weeks, following doxycycline treatment. Melanoma not developed up to 40 weeks.	[205,206]
Fish		Platyfish and swordtails Transgenic Zebrafish	Short generation time, large number of progenies, low cost, small housing Simple genetic manipulation, with transgenes or morpholinos injected into the embryo In Zebrafish, due to the transparency of their embryos, which develop externally, high-resolution visualization of transplanted fluorescent melanoma cells in vivo is possible with relative ease	[207,208,209,210,211,212,213,214,215,216]
Avian and other mammalian		Hamster Swine Horse Gray short-tailed opossum Chick embryo	In the chick embryo model: - the neural tube transplant used as a model for spontaneous neural crest migration: while there is spontaneous neural crest migration of melanoma cells, this is not the case for primary human melanocytes - the optic cup transplant used as a model for melanoma invasion - the rhombencephalon transplant used as a model for brain metastasis	[217,218,219,220,221,222,223,224,225,226,227,228,229]

Abbreviations: ARF: alternate reading frame; BRAF: v-raf murine sarcoma viral oncogene homolog B1; CDKN2A: cyclin-dependent kinase inhibitor 2A; Dct: dopachrome tautomerase; DMBA: 7,12-dimethylbenz(a)anthracene; GEM: genetically engineered models; GNAQ: guanine nucleotide-binding protein G(q) subunit alpha; GRM1: metabotropic glutamate receptor 1; HGF ⁄ SF: hepatocyte growth factor / scatter factor; NSG: non obese diabetic (NOD) SCID gamma; PDTXs: patient-derived tumor xenografts; PTEN: phosphatase and tensin homolog; RCAS/TVA: replication-competent avian sarcoma-leukosis virus long terminal repeat with splice acceptor/tumor virus A; RET: rearranged during transfection; RGP: radial growth phase; SCID: severe immuno-deficient; TPA: 12-o-tetradecanoylphobol-13-acetate; Tyr:Cre-ERT2: melanocyte-specific inducible Cre recombinase; UVR: ultraviolet rays; VGP: vertical growth phase.

**Table 3:** Nanomedicines developed in Italy in advanced pre-clinical characterization.

Nanosystem	Drug	Targeting Strategy	Stimuli Responsive	Animal Model	References
Leukosomes	Doxorubicin	Leukocyte membrane proteins		B16-F10 melanoma cells s.c. in C57BL/6 mice	[231]
Pyromellitic Nanosponges	Paclitaxel			B16-F10 melanoma cells s.c. in C57BL6/J mice	[232]
Solid Lipid Nanoparticles	Temozolomide			B16-F10 melanoma cells s.c. in C57BL6/J mice	[233]
Nanoemulsion (Intralipid^®^-IL)	Temozolomide rapamycin and bevacizumab			B16-F10 melanoma cells s.c. in C57BL6/J mice	[234]
Lipopolyplex vector	TRP2-mRNA/α- Galactosyl-ceramide	Size and Charge mediated-targeting to DC		B16-F10 melanoma cells s.c. in C57BL/6 mice	[235]
DOTAP- Liposomes	Clodronate	Size dependent uptake by TAM		B16-F10 melanoma s.c. in C57BL/6JOlaHsd mice B16-F10-luc melanoma cells iv in C57BL/6JOlaHsd mice, lung metastasis	[236]
CDNS and PLGA Np	ICOS-Fc			B16-F10 melanoma cells s.c. in C57BL/6J mice	[237]
PLA/PLGA Np	MART1 peptides (melanoma antigens)	Mannose (targeting DC)		Ret melanoma cells s.c. in of C57BL/6J B16-F10 melanoma cells s.c. in C57BL/6J mice	[238]
Phthalocyanine–gold Np conjugates	Zn(II)-C11Pc		PDT	B78H1 amelanotic clone of B16 melanoma cells s.c. in C57BL/6 mice	[239]
PEGylated gold Np	C11Pc		PDT	B78H1 amelanotic clone of B16 melanoma cells s.c. in C57BL/6 mice	[240]
Mesoporous Silica Np	Verteporfin		PDT irradiation	B16-F10 melanoma cells s.c. in C57BL/6J mice	[149]
Fibrin gels Loaded with a complex of DDP with HA and Chitosan	DDP	HA (CD44 and RHAMM)		B16 melanoma cells s.c. in nude mice SK-MEL-28 melanoma cells s.c. in NOD-SCID mice	[241]
Ferritin based Np	ADC: Ep1- cisplatin	Ep1-mediated melanoma targeting (melanoma specific antigen CSPG4)		Colo 38 human melanoma cells in CD1 nude mice	[242]
EXOs	TRAIL			INT12 melanoma cells (from a human melanoma specimen) s.c. in SCID mice	[243]
PCL and PEO Np	Docetaxel Zn(II)-phthalocyanine		PDT	A375 human melanoma cells s.c. in athymic Nu/Nu nude mice	[244]
ECFC-loaded chitosan-capped Gold Np			PA	A375-M6 melanoma cells (M6) were isolated in the laboratory from lung metastasis of SCID bg/bg mice	[245]
PEO-PDPA block copolymer polymersomes	Doxorubicin			Zebrafish embryos injected Red fluorescent B16 Melanoma Cells in the neural tube	[246]
Nanohybrids of catechin-gelatin conjugates incorporating carbon nanotubes	Catechin			WM266-4 human melanoma cell line microinjected into the yolk of zebrafish	[247]

Abbreviations: C11Pc: phthalocyanine disulphide; CDNS: β-cyclodextrin nanosponges; DC: dendritic cells; DOTAP: 1,2-dioleoyl-3-trimethylammonium-propane; DDP: cisplatin; ECFC: endothelial colony forming cells; EXOs: exosomes; HA: hyaluronic acid; ICOS: inducible co-stimulator; iv: intravenous; Np: nanoparticles; PCL: Poly(ε-caprolactone); PDPA: poly[2-(diisopropylamino)ethyl methacrylate]; PDT: photo-dynamic therapy; PA: photo-acoustic therapy; PEG: polyethylene glycol; PEO: Poly(ethylene oxide); PLA: poly-lactic acid; PLGA: poly-lactic-glycolic acid; s.c.: subcutaneous; TRAIL: Tumor necrosis factor-related apoptosis-inducing ligand.
